# Hospital-Wide Surveillance of Healthcare-Associated Infections as a Source of Information about Specific Hospital Needs. A 5-Year Observation in a Multiprofile Provincial Hospital in the South of Poland

**DOI:** 10.3390/ijerph15091956

**Published:** 2018-09-07

**Authors:** Małgorzata Kołpa, Marta Wałaszek, Anna Różańska, Zdzisław Wolak, Jadwiga Wójkowska-Mach

**Affiliations:** 1 State Higher Vocational School in Tarnów, St. Luke’s Provincial Hospital in Tarnów, 33-100 Tarnów, Poland; malgorzatakolpa@interia.pl (M.K.); mz.walaszek@gmail.com (M.W.); zdzich_w@interia.pl (Z.W.); 2 Department of Microbiology, Jagiellonian University, Polish Society of Hospital Infections, 31-121 Kraków, Poland; mbmach@cyf-kr.edu.pl

**Keywords:** hospital-acquired infections, surgical site infections, bloodstream infections, pneumonia, intensive care unit, neurosurgery, orthopaedics, Caesarean section, *Clostridium difficile*, rotavirus

## Abstract

Healthcare-associated infections (HAIs) are adverse complications of hospitalisation resulting in delayed recovery and increased costs. The aim of this study was an analysis of epidemiological factors obtained in the framework of constant, comprehensive (hospital-wide) infection registration, and identification of priorities and needs in infection control, both with regard to targeted surveillance, as well as preventative actions. The study was carried out according to the methodology recommended by the HAI-Net (Surveillance Network) coordinated by the European Centre for Disease Prevention and Control, in the multiprofile hospital in Southern Poland, between 2012 and 2016. A total of 159,028 patients were under observation and 2184 HAIs were detected. The incidence was 1.4/100 admissions (2.7/1000 patient-das of hospitalisation) and significantly differed depending on the type of the patient care: in intensive care units (ICU) 16.9%; in surgical units, 1.3%; non-surgical units, 1.0%; and paediatric units, 1.8%. The most common HAI was gastrointestinal infections (GIs, 28.9%), followed by surgical site infections (SSIs, 23.0%) and bloodstream infections (BSIs, 16.1%). The vast majority of GIs, BSIs, urinary tract infections, and incidents of pneumonia (PN) were detected in non-ICUs. As many as 33.2% of cases of HAI were not confirmed microbiologically. The most frequently detected etiologic agent of infections was *Clostridium difficile*—globally and in GI (49%). Comprehensive analysis of the results allowed to identify important elements of surveillance of infections, i.e., surveillance of GI, PN, and BSI not only in ICU, but also in non-ICU wards, indicating a need for implementing rapid actions to improve compliance with HAI prevention procedures.

## 1. Introduction

Starting from the 1980s, as a result of the Center for Disease Control and Prevention’s study of the efficacy of nosocomial infection control (SENIC study), many surveillance programs for healthcare-associated infections (HAIs) in the whole world are constructed on the basis of active and targeted surveillance. 

In the countries of the European Union, of which Poland is a member, European Centre for Disease Control and Prevention (ECDC) recommends active, targeted surveillance of selected infections in intensive care units, surgical site infections in certain surgical procedures, and *Clostridium difficile* infections. However, not all member states implement this recommendation actively. In Poland, such activities are very rare [1,2], which is why there are no national multicentre reports of overall rates of healthcare-associated infections. A change in the organizational culture in Polish hospitals to achieve better co-operation between infection control practitioners and clinical staff is required for the effective prevention and control of HAI in our country. However, such a different situation is also the case in those countries which are only beginning the implementation of infection control in their hospitals—as confirmed by ECDC reports [3,4], in which no data is available for many Eastern Bloc countries.

However, on the other hand, such strict limitation of targeted surveillance can, in a significant way, direct the attention of infection control teams only to specific patient populations, while certain patient populations should entail surveillance of other forms of infections as well, depending on the type of ward [5], and also, surgical site infections in the surgical procedures not covered by ECDC recommendations. In order to detect all HAIs and all needs and limitations to HAI prevention, hospital-wide HAI surveillance (at least periodically) is required. Such an approach allows an in-depth analysis of the hospital epidemiological situation and the hospital’s own data analysis is the basis for creating the scope of infection control programmes.

Results obtained in the framework of infection registration should also be conveyed as feedback to the staff of the wards, as a starting point for the introduction or modification of procedures preventing infections, including environmental hygiene [6], and especially hand hygiene. The results of Polish research concerning the knowledge, perception, and practice, as regards hand hygiene, indicate a very low compliance with this procedure by healthcare workers [7,8].

The aim of this study is an analysis of epidemiological factors obtained in the framework of constant, comprehensive (hospital-wide) infection registration, and identification of priorities and needs in infection control, both with regard to targeted surveillance, as well as preventative actions. The continuous surveillance involved all clinical forms of HAIs, taking into consideration HAIs in all departments of a multiprofile hospital, spanning 5 years. 

## 2. Material and Methods

Infection surveillance was conducted in 2012–2016 in St. Luke’s Provincial Hospital in Tarnów, Poland, the non-teaching secondary care hospital, and one of the biggest institutions in the south of Poland, with 760 beds. At the hospital, there is one 9-bed intensive care unit (ICU), treating medical, surgical and trauma patients, nine surgical units, seven medical assessment units, and a children and newborn unit. Patients are admitted to the hospital both electively, as well as in an emergency through the Emergency Unit. Emergency admissions constitute around 40% of all admissions. In the hospital, there is a microbiological laboratory performing more than 35,000 clinical material tests, on average, a year. All materials taken from the gastrointestinal tract infections are tested for *Shigella* spp., *Salmonella* spp., rotavirus, norovirus, adenovirus, *Clostridium difficile* (CD), and a general stool culture is performed. *Clostridium difficile* infection (CDI) was confirmed by a *Clostridium difficile* cassette test (TechLab, Blacksburg, VA, USA).

The Infection Control Team consists of a doctor who is 1/3 full-time equivalent, and 4 full-time epidemiological nurses. Active surveillance of infections was introduced in the hospital in 2001, and the results concerning selected, specific epidemiological data have already been published. The authors’ experiences concerning the previous years have already been partially discussed, however, they concerned individual patient types without taking into account the context of operation of the entire hospital: different types of infections and the patient populations treated [9,10,11,12,13]. This analysis included hospital-wide results of the surveillance of all infection types in all units, in order to identify areas demanding special attention and activities of infection control team. 

The hospital participates in a voluntary nationwide system of active registration of all types of healthcare-associated infections described by ECDC, consistent with the methodology of the European HAI-Net. The Healthcare-Associated Infections Surveillance Network (HAI-Net) is a European network for surveillance of HAIs [14]. The network is coordinated by the European Centre for Disease Prevention and Control (ECDC), an EU agency established in 2005 and aimed at strengthening Europe’s defences against infectious diseases. Participation in HAI-Net is voluntary and confidential. HAIs were diagnosed (with or without the microbiological confirmation) and classified based on the uniform definitions issued by the ECDC, in accordance with the decision of the European Commission 2002/253/EC laying down case definitions for reporting communicable diseases to the community network (https://eur-lex.europa.eu/legal-content/EN/TXT/?uri=CELEX%3A32012D0506). The study excluded patients whose hospital stay was shorter than 2 days and patients who showed symptoms of infection within 2 days of admission. All surgical site infections (SSIs) were detected during the first hospital stay of a given patient, when readmitted due to infection, or in the course of a post-discharge follow-up appointment at the outpatient clinic.

The following epidemiological measures were used: the cumulative incidence and the density incidence. The cumulative incidence was defined as the number of infection episodes divided by the number of admitted patients and incidence density—divided by the number of person-days of hospitalisation. Statistical analysis of the data was performed using SPSS software (SPSS—Statistical Package for the Social Sciences, STATISTICS 24, Armonk, NY, USA). For statistical analysis of ordinal or dichotomous data, information on the number and percentage of individuals was used. The mean, median (Me), standard deviation (SD), 95% confidence interval (95% CI), minimum, and maximum were calculated. For ordinal and nominal variables, Pearson’s chi-square (χ^2^) test was used. Statistical significance was assumed at a level of *p* < 0.05.

The use of data was approved by the Bioethical Committee of the Jagiellonian University (No. KBET/122.6120.118.2016 from 25.05.2016). All data entered into the electronic database and analysed in this study were previously anonymised.

## 3. Results

The total number of patients in the study was 159,028, and average patient age was 56, SD 26.5; in the ICU it was 58, SD 18.6; in surgical units was 59, SD 20.6. The oldest patients were hospitalised in non-surgical units; average age was 52, SD 33.5. 

There were 2184 HAIs in total, and the incidence rate was 1.4% and incidence 2.7 per 1000 patient-days of hospitalisation (pds. The incidence significantly varied depending on the type of ward: ICU demonstrated the highest incidence, 16.9% (*n* = 203); while in the surgical units it was 1.3% (*n* = 1112); in non-surgical departments, 1.0% (*n* = 623); and in paediatric units, 1.8% (*n* = 246) (Table 1).

The most frequently detected HAI types were gastrointestinal tract infections (GIs) constituting 28.3% of all HAIs; also, the incidence of this type of infection was the highest, both in ICU and non-ICUs. In surgery units, the GI incidence was not much lower than the one associated with SSIs (Table 2). The incidence of *Clostridium difficile* infection (CDI) was 3.7/10,000 pds, and in the ICU: 13/10,000 pds. 

The second group of infections was surgical site infection (SSI), with an incidence rate of 1.4% (per 100 operations). The majority of SSIs were found in surgical oncology (e.g., colon surgery) and neurosurgery (e.g., craniotomy) departments, but the highest incidence was associated with the rarely performed procedure of ventricular shunt implantation, and it amounted to 12.2% (Table 3). In surgical units, SSI amounted to only 44.4% of all forms of HAI. Among the remaining ones, the dominant infections were bloodstream infections (BSIs), urinary tract infections (UTIs), and pneumonia (PN). The percentage of various etiologic agents differed insignificantly in various types of units, but in 25.1% of cases, the infection was not confirmed microbiologically (Table 4). *Staphylococcus aureus* and *Escherichia coli* were the predominant causes of SSI, and aetiology was not confirmed in 13.6% of cases; in 52 cases of SSI, the test result was negative, in 7 cases, no material was taken for examination (Table 4).

Severe infections, i.e., BSIs and PN (which constituted 15.8% and 11.0% of all HAIs, respectively) with the incidence rate in the ICU of 6.0% and 5.5%, respectively, mainly concerned the patients from the remaining units: in the ICU, they were only 20.9% of all BSIs and 27.4% of all PN cases (Table 2). A significant amount of PN cases concerned neurological and neurosurgical patients (Table 2) and mechanical ventilation was carried out in 5 and 13 patients, respectively.

In BSIs, *Staphylococcus aureus* was often isolated, which was similar to PN; however, *Acinetobacter baumannii* was most frequently isolated for PN. UTIs generally concerned the patients from internal units, although the incidence was significantly higher in ICU (Table 2). The main etiologic agent was *Escherichia coli*. 

Attention should be drawn to the high HAI morbidity in the paediatric wards, where the stays were relatively short, with a median of 3 days, average 3.6 (95% CI 3.4–3.9), SD 1.5, but the incidence was significantly higher than in other medical treatment wards (for adults). Out of all 146 cases of HAI, 128 cases (87.7%) were GIs: rotaviruses, 77 (60.2%); noroviruses, 21 (16.4%); and adenoviruses, 2 (1.6%). Detailed data on incidence rates according to aetiology or GIs are presented in Table 5.

Generally, HAIs are predominantly caused by *Staphylococcus aureus* and *Escherichia coli*, which amounted to 37.6% in total, however, with regard to microbiological surveillance, it should be especially emphasised that 33.2% of HAI cases were not microbiologically confirmed.

## 4. Discussion

The research presented is one of the few studies available in Poland that deals with the incidence rates of HAIs in many areas of medicine. Currently, it is assumed that surveillance of infections in hospitals should be carried out on the basis of priorities, in order to focus the surveillance activities on these elements that really require intervention. However, to get to know a particular hospital, its needs and shortcomings regarding the prevention of infections—implementation of infection control—could begin with a full observation encompassing all hospital functions, i.e., hospital-wide surveillance. That is why it was taken at our hospital, and the data presented indicate a number of important issues that require action in the field of infection control.

The incidence rates of *Clostridium difficile* infections and viral gastrointestinal infections obtained in our study put them in first place amongst all the infections detected in the hospital studied. The fact that GI was dominant over others can indicate a better detectability of this form of infection in the test, and its better diagnostics, and hence, ease in detecting this type of infection, although around ¼ of cases were not confirmed microbiologically. Yet, it can also be the result of growing difficulties in cooperation between the members of the infection control team, medical staff, and administration, regarding recognition of different HAIs.

Overall, CDI incidence seems to coincide with the values obtained in many European countries, which reported incidences ranging from 2.8 to 4.4/10,000 pds in 2016 [15]. In our study, the data for the entire hospital was 3.7/10,000 pds, and correspond to other reports from Poland. In the research by Pituch et al. [16] carried out in Poland in 2011–2013, the incidence of CDI was 8/10,000 pds, on average, but in hospitals at our referral level, it was 5/10,000 pds. In addition, CDI incidence in ICU, where the value obtained amounted to 1.3/1000 pds, was much lower than in one from other Polish ICUs, e.g., Ziółkowski et al. where CDI incidence in ICU was 10.6/10,000 pds [17]. 

Viral GIs were a significant problem in the paediatric department, similarly to the work by Jackowska and Pawlik [18], where the proportion of viral healthcare-associated infections amounted to 96% of all infections. A comparable report from Quebec indicates a proportion of rotaviruses different from the studied one—20.4%, especially for noroviruses, 25.5%, in the aetiology of GIs [19] (in our study, it was respectively 60.2% and 16.4%), and concerns the population in which, for a couple of years now, vaccines against both viruses have been reimbursed—in Poland, these vaccinations are patient-paid and only recommended.

Where do such big problems with GIs and high-infectivity infections stem from, not only in the hospital studied, but also in the whole of Poland? It may be the result of the immaturity of the surveillance system and insufficient hand hygiene in Polish healthcare facilities [20]. Unfortunately, other reports from Poland also indicate poor compliance with the hand hygiene procedure, which is one of the important risk factors for healthcare-associated gastrointestinal tract infections, especially those caused by viruses [7,8].

In the present study, the second place among HAIs was occupied by SSIs, especially in neurosurgery, however, the morbidity observed corresponded to the literature data [3,21]. Nevertheless, all incidents of SSIs signal a significant dominance of deep incisional cases, which probably directly stems from the lack of post-discharge surveillance; therefore, superficial incisional cases, diagnosed in outpatient settings and not requiring hospitalisation, were not subject to registration or, consequently, analysis in this study. Similar results in other Polish studies were reported by Dubiel et al. in the field of thoracic surgery [5] or Różańska etc. in obstetrics [22]. A substantial problem for patients of surgical wards in the hospital studied turned out to be other HAIs, which amounted to over a half of all cases, which is a clue pointing to the fact that the basic problem in HAI prevention is general medical care and that limiting surveillance to SSI only would worsen the patients’ prognoses greatly. In studied surgical units, targeted surveillance cannot be solely limited to SSIs. This is of paramount importance in neurosurgery, where many patients are admitted as emergencies, and where the predominant reasons for the occurrence of nosocomial pneumonia are brain diseases and injuries resulting in the need for surgery in this area, and are associated with the risk of loss or impairment of important functions of the nervous system. Similar observations in Polish were recorded by Dubiel et al. [5] who, in patients undergoing chest surgery, demonstrated that there is a need to submit the subjects to active targeted surveillance of pneumonia, and not only SSIs.

Unfortunately, this issue, i.e., the proportion of severe infections, secondary BSI and PN, mainly concerned the patients hospitalised outside the ICU, and a significant proportion of PN cases involved neurological and neurosurgical patients. In the neurology departments, the main determinants of the occurrence of pneumonia are strokes, which is confirmed by Kumar et al. [23] who indicate that the incidence in patients with acute stroke was 34%. Control of these forms of infections should become an absolute priority for the local infection control team, both in regard to the prevention as well as diagnosis—ca. 60% of cases of these infections were not confirmed microbiologically. This may have consequences in the incidence of CDI, since long-term antibiotic therapy (especially empirical) may result in intestinal dysbiosis, and increase the risk of CDI [24,25]. Equally disturbing are the data concerning the remaining HAI forms, mainly diagnosed outside of the ICU, especially in internal units—it is there that the problem may appear with recognising such rarer infections, or lack of knowledge of the rules of their prevention and control, e.g., drawing material for microbiological testing.

On the other hand, incidence in ICU remains at the expected level. Data on BSI incidence from a Polish multicentre study in 2013–2015 give a virtually identical value [26], although an ECDC report [11] declares this value to be 3% in 2014 but, at the same time, major differences were indicated between the individual countries. These differences may be determined by surveillance sensitivity, with particular emphasis on microbiological testing in individual countries [4]. This was similar in the case of PN: the value obtained corresponds to data from other Polish ICUs [27]. This situation indicates that it was relatively easy to introduce surveillance of infections in this particular unit, and pinpoints the priority element: CDI, which is, fortunately, an attainable goal [28].

The research results presented are one of the few studies from Poland based on the method of active and continuous HAIs surveillance. They can constitute a basis to compare the epidemiological situation in different hospitals which, consequently, may be conducive to taking action for effective HAI prevention. However, the Polish HAIs surveillance system has a short 20-year tradition [29,30], and a review of the literature indicates that it does not function optimally. 

There are some limitations of the present study. Firstly, the research involves only one centre. Secondly, in the period studied, despite participation in the multiprofile programme, the infection registration method was not validated, hence, its sensitivity is not known in this particular case. Low HAI incidence, especially with regard to SSIs, may be proof of poor sensitivity in the surveillance associated with lack of post-discharge registration [23], or ineffectiveness of the prevention programs implemented, both at the local and national levels [1].

However, as is clear from our research, the surveillance system can also function efficiently when its sensitivity is far from perfect; provided that it is stable in time and allows the detection of changes with regard to incidence. The strength of this study is complex, hospital-wide surveillance, owing to which it was possible to identify the areas requiring increased infection prevention and registration. 

## 5. Conclusions

Selecting priorities in infection control should incorporate the local epidemiological situation, therefore, it seems reasonable to implement active surveillance of infections with the use of hospital-wide surveillance, to detect all HAIs and all limits to HAI prevention. The overall HAI incidence in the hospital investigated may point to low sensitivity of the infection detection system, however, an in-depth analysis of HAI types demonstrates that the system functions efficiently. Intervention is required with regard to the dominant GIs, also in ICU, and a high percentage of non-surgical infections in patients undergoing surgery and epidemiology of secondary BSI outside ICU.

## Figures and Tables

**Table 1:** Cumulative incidence rates and incidence density rates in different types of hospital units.

Studied Units	Admissions *n* = 159,028	Patient-Days *n* = 821,088	HAI *n* = 2184	Incidence Rates	
				Cumulative Per 100 Admissions	Density Per 1000 pds
Intensive care					
Intensive care unit	1201	9211	203	16.9	22.0
Surgical units					
Surgical oncology	3353	13,225	99	3.0	7.5
Neurosurgery	10,878	58,443	303	2.8	5.2
General surgery	13,822	68,502	287	2.1	4.2
Urology	8243	33,605	169	2.1	5.0
Orthopaedic—trauma	7627	47,158	146	1.9	3.1
Paediatric surgery	8308	23,000	61	0.7	2.7
Gynaecology and obstetrics	13,746	56,077	36	0.3	0.6
Otolaryngology	6242	22,031	7	0.1	0.3
Ophthalmology	11,648	24,839	2	0.1	0.1
**Subtotal**	**83,867**	**346,880**	**1110**	**1.3**	**3.2**
Non-surgical units					
Internal diseases and nephrology	7524	55,845	289	3.8	5.2
Rehabilitation	2803	71,370	72	2.6	1.0
Neurology	11,808	73,113	121	1.0	1.7
Radiotherapy	3485	46,761	34	1.0	0.7
Internal diseases and acute poisoning	10,083	52,511	42	0.4	0.8
Cardiology	13,943	57,187	53	0.4	0.9
Oncology	10,319	32,279	14	0.1	0.4
**Subtotal**	**59,965**	**389,066**	**625**	**1.0**	**1.6**
Paediatric units					
Neonatal	5170	50,770	100	1.9	2.0
Paediatric	8825	25,161	146	1.7	5.8
**Subtotal**	**13,995**	**75,931**	**246**	**1.8**	**3.2**
**TOTAL**				**1.4**	**2.7**

Abbreviation: HAI—Healthcare-associated infection; pds—patient-days of hospitalisation.

**Table 2:** Incidence of healthcare-associated infections (HAIs) by major and specific site of infection in 2012–2016.

All HAIs, N-203	ICU		Non-Surgical Units		Surgical Units		Total		Share in the Total Infection Pool (%)
	*n*	Incidence (%)	*n*	Incidence (%)	*n*	Incidence (%)	*n*	Incidence (%)	
Gastrointestinal infection (GI)	21	1.75	397	0.54	219	0.26	619	0.39	28.3
*Clostridium difficile* infection	12	1.00	168	0.23	123	0.15	303	0.19	
gastroenteritis	9	0.75	211	0.29	96	0.11	316	0.20	
Surgical site infection (SSI)							493	0.31 *	22.6
Bloodstream infections (BSI)	72	6.00	116	0.16	157	0.19	345	0.22	15.8
Primary BSI catheter-related	14	1.17	38	0.05	26	0.03	78	0.05	
Primary BSI of unknown origin	33	2.75	42	0.06	61	0.07	136	0.09	
Primary BSI	11	0.92	4	0.01	26	0.03	41	0.03	
Secondary BSI	14	1.17	32	0.04	44	0.05	90	0.06	
Urinary tract infection (UTI) **	19	1.58	163	0.22	107	0.13	289	0.18	13.2
Pneumonia (PN)	66	5.50	66	0.09	109	0.13	241	0.15	11.0
Skin and soft tissue infection	7	0.58	53	0.07	20	0.02	80	0.05	3.7
Ear, eye, mouth	2	0.17	35	0.05	1	0.00	47	0.03	2.2
Neonatal infection			40	0.05		0.00	40	0.03	1.8
Lower respiratory tract infection	3	0.2	7	0.01	5	0.01	15	0.01	0.7
Central nervous system infection					8	0.01	8	0.01	0.4
Reproductive tract infection					7	0.01	7	0.00	0.3
**Total**									**100%**

* Incidence per 100 surgeries was 1.4%. Intensive care unit (ICU); ** all types of UTI: associated and not associated with catheterization.

**Table 3:** Characteristics of surgical site infections (SSIs) and incidence rates associated with procedure types.

Type of SSIs *n* = 493		
SSI-D: Deep incisional *n* = 346	70.2%	
SSI-O: Organ/space *n* = 69	14.0%	
SSI-S: Superficial incisional *n* = 78	15.8%	
Total	100%	
**Type of Procedures**	**No. SSI**	**Incidence (%)**
Ventricular shunt implantation *n* = 115	14	12.2
Spinal immobilization *n* = 953	6	0.6
Knee prosthesis *n* = 271	3	1.1
Hip prosthesis *n* = 1016	16	1.6
Open reposition of long bone fractures *n* = 1079	15	1.4
Oncological breast surgery *n* = 606	4	0.7
Oncological colon surgery *n* = 749	15	2.0
Laminectomy *n* = 2032	14	0.7
Craniotomy *n* = 1049	19	1.8
Appendectomy *n* = 384	4	1.0
Cholecystectomy *n* = 1872	5	0.3
Caesarean sections *n* = 2083	2	0.1

**Table 4:** Aetiology of different types of healthcare-associated infections (HAIs), without gastrointestinal infection.

Microorganism	SSI	BSI	UTI	PN	OTHER	Total
	*n* (%)	*n* (%)	*n* (%)	*n* (%)	*n* (%)	*n* (%)
**Gram-positive cocci**						
*Staphylococcus aureus*	102 (23.5)	74 (24.7)	7 (2.5)	29 (19.7)	25 (18.2)	237 (18.3)
*Coagulaase-negative staphylococci*	28 (6.5)	69 (23.0)			26 (19.0)	123 (9.5)
*Enterococcus* spp.	46 (10.6)	28 (9.3)	32 (11.6)	2 (1.4)	5 (3.6)	113 (8.7)
*Streptococcus* spp.	9 (2.1)	4 (1.3)	1 (0.4)	1 (0.7)	5 (3.6)	20 (1.5)
**Enterobacteriaceae**						
*Escherichia coli*	86 (19.8)	36 (12.0)	92 (33.3)	13 (8.8)	22 (16.1)	249 (19.3)
*Klebsiella* spp.	19 (4.4)	21 (7.0)	40 (14.5)	14 (9.5)	12 (8.8)	105 (8.1)
*Enterobacter* spp.	27 (6.2)	12 (4.0)	8 (2.9)	7 (4.8)	4 (2.9)	58 (4.5)
*Proteus* spp.	20 (4.6)	6 (2.0)	21 (7.6)	8 (5.4)	4 (2.9)	59 (4.6)
*Serratia* spp.	4 (0.9)	5 (1.7)		1 (0.7)		10 (0.8)
**Non-fermenting Gram-negative bacteria**						
*Acinetobacter baumannii*	31 (7.1)	24 (8.0)	12 (4.3)	54 (36.7)	14 (10.2)	135 (10.4)
*Pseudomonas aeruginosa*	28 (6.5)	1 (0.3)	17 (6.2)	4 (2.7)	6 (4.4)	56 (4.3)
*Morganella morganii*	13 (3.0)	1 (0.3)	6 (2.2)	5 (3.4)	1 (0.7)	26 (2.0)
Other bacteria	15 (3.5)	4 (1.3)	5 (1.8)	3 (2.0)	2 (1.5)	29 (2.2)
*Candida* spp.	6 (1.4)	15 (5.0)	35 (12.7)	6 (4.1)	11 (8.0)	73 (5.6)
Total	434 (100)	300 (100)	276 (100)	147 (100)	137 (100)	1293 (100)
Negative result	52 (12.0)	39 (13.0)	9 (3.3)	52 (35.4)	65 (47.4)	348 (26.9)
Not collected	7 (1.6)	11 (3.7)	4 (1.4)	41 (27.9)	15 (10.9)	82 (6.3)

SSI—surgical site infections, BSI—bloodstream infections, UTI—urinary tract infections, PN—pneumonia.

**Table 5:** Incidence of gastrointestinal infections according to aetiology.

Microorganism	Gastrointestinal Infection (GI)				
	*n*	%	GI/100 Admissions	GI/1000 pds	GI/10,000 pds
**Gram-positive other**					
*Clostridium difficile*	307	49.2	0.2	0.4	3.7
**Viruses**					
*Rotavirus*	103	16.5	0.1	0.1	1.3
*Adenovirus*	5	0.8	0.0	0.0	0.1
*Norovirus*	46	7.4	0.0	0.1	0.6
**Total**	**461**	**73.9**	**0.3**	**0.6**	**5.6**
Negative result	131	21.0	0.1	0.2	1.6
Not collected	32	5.1	0.0	0.0	0.4

pds—patient-days of hospitalisation (*n* = 821,088), number of hospitalisations (*n* = 159,028).

## Abbreviations

HAI	Healthcare-associated infection
ICU	intensive care unit
GI	gastrointestinal infection
BSI	bloodstream infection
PN	pneumonia
UTI	urinary tract infection
SSI	surgical site infection
SPSS	Statistical Package for the Social Sciences
Me	median
SD	standard deviation
95% CI	95% confidence intervals
ECDC	European Centre for Disease Prevention and Control
HAI-Net	Healthcare-associated Infections Surveillance Network
CI	cumulative incidence
pds	patien-days of hospitalization
ICD	International Classification of Diseases
CDI	Clostridium difficile infection
